# γ-Valerolactone-Enabled Mild Methanolysis of Waste Polyethylene Terephthalate for Efficient Chemical Recycling

**DOI:** 10.3390/polym17111458

**Published:** 2025-05-24

**Authors:** Zhao Ding, Xing Cao, Xin-Yu Hao, Yan-Peng Ni

**Affiliations:** Institute of Functional Textiles and Advanced Materials, Qingdao Key Laboratory of Flame-Retardant Textile Materials, National Engineering Research Center for Advanced Fire-Safety Materials D & A (Shandong), College of Textiles & Clothing, Qingdao University, Qingdao 266071, China; 17791484080@163.com (Z.D.); caoxingmini1016@163.com (X.C.); haoxinyu010724@163.com (X.-Y.H.)

**Keywords:** waste PET, chemical recycling, methanolysis, γ-valerolactone (GVL), bio-based solvent

## Abstract

To tackle growing resource and environmental challenges, closed-loop chemical recycling of waste PET is gaining significant attention. Methanolysis demonstrates significant industrial potential due to the ease of separation and purification of its depolymerization product, dimethyl terephthalate (DMT). However, conventional methanolysis processes for PET typically require harsh conditions (>200 °C and 2–4 MPa), highlighting the need for more efficient and milder methods. In this work, leveraging Hansen’s solubility parameter theory, a bio-based solvent gamma-valerolactone (GVL) was introduced to construct a binary mixed solvent system, enabling highly efficient depolymerization of PET. Through systematic optimization of reaction conditions, an in-depth analysis of the effects of various factors on depolymerization efficiency and kinetics was conducted. The incorporation of GVL markedly enhanced the compatibility between the solvent and PET, thereby significantly improving depolymerization efficiency while effectively lowering the reaction temperature and pressure. Complete depolymerization of PET can be achieved within 2 h at 150 °C under a pressure of 0.9 MPa, with a DMT yield of up to 97.8%. This GVL/methanol depolymerization system exhibits higher efficiency, milder reaction conditions, and substantial advantages in terms of environmental impact and energy consumption indicators. By using the renewable bio-based solvent GVL, this technology aligns with the core principles of green chemistry and provides an efficient, feasible, and innovative pathway for sustainable closed-loop PET recycling.

## 1. Introduction

Polyethylene terephthalate (PET) has gained extensive applications across various industries, including textiles, electronics, construction, and food packaging, owing to its exceptional physical and chemical properties, as well as its cost-effectiveness [1,2]. However, the rapid increase in PET waste generation poses significant environmental challenges. PET waste, once discarded after use, is highly resistant to degradation in natural environments and can contribute to microplastic pollution [3,4]. Furthermore, as a petroleum-based polymer derived from non-renewable resources, PET requires substantial resources and energy for its production [5,6,7]. Current recycling statistics indicate that PET recycling efficiency remains low, with only a small fraction of PET products being effectively recycled. The majority of PET waste is either incinerated or landfilled, which not only leads to resource wastage but also exacerbates environmental pollution [8]. Confronted with the surge in plastic waste and inefficient recycling systems, developing efficient PET recycling technologies has become an urgent requirement to mitigate plastic pollution and resource scarcity [9,10].

The current strategies for PET recycling primarily encompass physical and chemical approaches. Physical recycling predominantly employs mechanical processing techniques to transform post-consumer PET into secondary raw materials or recycled products [11]. Despite its notable advantages, such as low energy consumption, straightforward operation, and favorable economic and environmental performance, this method’s applicability is constrained, as it is suitable only for relatively pure PET products and predominantly associated with downcycling [12]. Consequently, the quality and value of the recycled products are generally lower. In contrast, chemical recycling focuses on fully depolymerizing PET into monomers such as terephthalic acid (TPA), dimethyl terephthalate (DMT), bis(2-hydroxyethyl) terephthalate (BHET), or partially depolymerizing it into oligomers, which can serve as raw materials for PET production or other high-value materials synthesis. Chemical recycling offers a promising solution for closing the material cycle in PET lifecycle management while addressing critical sustainability challenges [13,14,15,16]. The prevailing chemical recycling methods include hydrolysis, alcoholysis, and ammonolysis reactions [17,18,19]. Among these, alcoholysis has demonstrated the most extensive industrial applications, particularly through glycolysis and methanolysis processes [20]. Notably, methanolysis exhibits significant advantages over other alcoholysis variants, especially in facilitating the effective separation and purification of depolymerization products DMT [21]. This approach enables the direct recovery of high-purity DMT via established purification protocols, thereby serving as an optimal raw material for the preparation of high-quality recycled PET materials through repolymerization [22,23].

Conventional methanolysis processes for PET depolymerization generally demand severe conditions, including high temperatures (180–220 °C) and elevated pressures (2–4 MPa), to break down PET into dimethyl terephthalate (DMT) and ethylene glycol (EG) [15,24]. This results in relatively low depolymerization efficiency and substantial energy consumption. To enhance depolymerization efficiency, several advanced techniques have been developed, such as high-efficiency depolymerization catalysts, supercritical methanol processes, microwave-assisted processes, and solvent-assisted depolymerization methods. For instance, Laldinpuii et al. [25]. developed a green catalytic system using bamboo leaf ash (BLA) as a heterogeneous catalyst for methanolysis. Under optimal conditions (200 °C, 2 h), it achieved 78% DMT and 76% EG yields. Tang et al. [23]. developed a MgO/NaY catalyst that demonstrates superior catalytic performance attributed to its strong alkaline active sites, high specific surface area, and well-developed pore structure. At a methanol-to-PET ratio of 6, a 4 wt% catalyst dosage, and 30 min at 200 °C, the PET conversion and DMT yield were 99% and 91%, respectively.

In addition to the development of efficient catalysts, a variety of process intensification techniques have also been employed to enhance PET depolymerization. Among these, supercritical methanolysis operates under elevated conditions (260–270 °C, 9–11 MPa), significantly accelerating reaction kinetics while achieving DMT yields approaching 90% [26]. Microwave-assisted depolymerization can achieve complete depolymerization of PET within 10 min at 210 °C by increasing energy input while maintaining a product yield of 95% (DMT and EG) [27]. Despite these advances, which have significantly reduced reaction times from hours to minutes, supercritical and microwave-assisted methods still necessitate stringent reaction conditions. This imposes rigorous demands on reaction vessel design, especially concerning pressure management and operational safety, thereby considerably restricting their scalability for industrial applications. Solvent-assisted depolymerization has emerged as a highly effective approach for promoting efficient PET breakdown under mild operational conditions [28,29,30]. This strategy eliminates the requirement for extreme temperature and pressure parameters while maintaining high catalytic efficiency. Recent studies have demonstrated that several solvents, such as gamma-valerolactone and tetrahydrofuran, which exhibit solubility parameters closely aligned with those of PET, hold the potential to significantly enhance the hydrolysis or ethylene glycol depolymerization of PET [31,32,33]. For the methanolysis method of PET, although adding cosolvents can improve the efficiency of the depolymerization reaction, the introduction of cosolvents may also bring new challenges. For example, although the solubility parameter of THF is relatively close to that of PET, its low boiling point imposes higher requirements on equipment under high-temperature reaction conditions, which increases experimental safety hazards. Therefore, the selection of cosolvents should not solely rely on solubility parameters, but should also comprehensively consider their availability, safety, and interactions with PET.

**Scheme 1:**
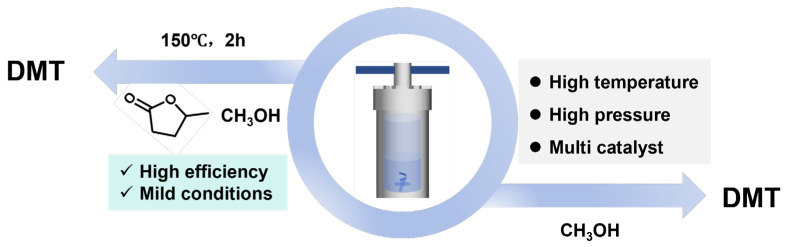
Methanol hydrolysis of PET assisted by GVL.

In this work, As shown in Scheme 1 an efficient mixed-solvent system for PET methanolysis was developed by utilizing gamma-valerolactone (GVL), a bio-based solvent, as a co-solvent. Through systematic investigation using the controlled variable method, the effects of key operational parameters, including solvent-to-additive ratio, reaction temperature, catalyst concentration, and reaction time, on depolymerization efficiency were comprehensively evaluated. Furthermore, the kinetic characteristics of the depolymerization reaction were thoroughly analyzed. Additionally, a quantitative assessment of the environmental impact of the associated processes was conducted. It is encouraging that the introduction of GVL offers dual advantages: compared with traditional high-temperature processes (e.g., 210–270 °C), it not only significantly enhances depolymerization efficiency but also decreases the required reaction temperature by 40–60 °C, thereby accelerating reaction kinetics. Under optimized conditions, this process achieves complete PET conversion within 2 h at 150 °C.

## 2. Experiment Section

### 2.1. Materials

Post-consumer colorless and transparent PET bottle bodies were washed, air-dried, and cut into 3 × 3 mm square samples. γ-Valerolactone (GVL, ≥98% purity) and zinc acetate dihydrate (Zn(OAc)_2_·2H_2_O, ≥99% purity) were procured from Macklin Co., Ltd. (Shanghai, China). Methanol (MeOH, ≥99. 5% purity) was acquired from Tianjin Fuyu Fine Chemical Co., Ltd. (Tianjin, China). All reagents were used without further purification.

### 2.2. Solvent Selection for PET Depolymerization

Solubility parameters serve as a fundamental criterion for solvent selection. Solvents and solutes exhibit enhanced mutual miscibility when their solubility parameters are closely matched, with solutes demonstrating preferential dissolution in solvents possessing similar solubility characteristics. The Hansen solubility parameters (HSP), formulated through Hansen’s three-dimensional parameter model (dispersive δ_D_, polar δ_P_, and hydrogen bonding H interactions), have gained widespread adoption for predicting solvent selectivity. This approach enables quantitative evaluation of solvent compatibility by analyzing the geometric distance (R_a_) between solute and solvent HSP values in three-dimensional space, where lower R_a_ values correlate with superior dissolution performance [34].

The Hansen solubility parameters (HSP) methodology has been widely adopted for solvent selection, grounded in the theoretical postulation that the total cohesive energy (E_t_) of a pure compound comprises three distinct interactional components. This foundational principle is mathematically expressed in Equation (1):

(1) $$E_{t}=E_{d}+E_{p}+E_{h}$$

Herein, E_d_, E_p_, and E_h_ correspond to non-polar (dispersive), polar (dipole–dipole and dipole-induced dipole), and hydrogen bonding/other associative interactions, respectively. Each energy component is normalized by the molar volume (V_m_), mathematically expressed in Equation (2):

(2) $$\frac{E_{t}}{V}=\frac{E_{d}}{V}+\frac{E_{p}}{V}+\frac{E_{h}}{V}=\;\delta_{d}^{2}\;+\delta_{p}^{2}+\delta_{h}^{2}$$

Herein, V represents the molar volume (units: mol·m⁻^3^). The Hansen solubility parameters are defined as: δ_d_: dispersive solubility parameter; δ_p_: polar solubility parameter; δ_h_: hydrogen bonding solubility parameter.

Consequently, the total solubility parameter (δ_t_) is calculated via Equation (3):

(3) $$\delta_{t}=\sqrt{\delta_{d}^{2}+\;\delta_{p}^{2}+\delta_{h}^{2}}$$

The Hansen solubility parameters (δ_d_, δ_p_, and δ_h_) can be obtained from published literature databases or calculated through the widely recognized group contribution method (Hoftyzer–Van Krevelen):

(4) $$\delta_{d\;}=\frac{\sum_{i}F_{\mathrm{di}}}{\sum_{i}V_{i}}$$

(5) $$\delta_{p}=\frac{\sqrt{\sum_{i}F_{\mathrm{pi}}^{2}}}{\sum_{i}V_{i}}$$

(6) $$\delta_{h\;}=\sqrt{\frac{\sum_{i}E_{\mathrm{hi}}}{\sum_{i}V_{i}}}$$

### 2.3. PET Depolymerization and Monomer Recovery Process

The PET depolymerization process was conducted as follows: A mixture solution (3–10 g) of γ-valerolactone (GVL)/methanol (MeOH) with a mass ratio ranging from 7:3 to 3:7 was prepared and loaded into a reactor. PET samples (1 g) were then added to the solution, followed by the introduction of a stoichiometric amount of catalyst (0.05–1 wt% relative to PET mass). The reaction was carried out under controlled conditions (140–180 °C) with continuous stirring for 0–200 min to facilitate efficient depolymerization. After completion, lower the temperature and filter the insoluble substances. Subsequently, add it to hot methanol, filter the unreacted PET, then lower the temperature of the filtrate to filter out DMT crystals, and dry under vacuum to obtain purified DMT.

The degradation rate of PET and the yield of DMT were quantitatively assessed using Equations (7) and (8), respectively:

(7) $$\mathrm{PET}\;\mathrm{degradation}\;\mathrm{rate}\left(\%\right)=\frac{m_{\mathrm{PET}}\;-\;m_{\mathrm{PET-r}}}{m_{\mathrm{PET}}}\;\times \;100$$

(8) $$\mathrm{DMT}\;\mathrm{yield}\left(\%\right)=\frac{\frac{m_{\mathrm{DMT}}}{M_{\mathrm{DMT}}}}{\frac{m_{\mathrm{PET}}}{M_{\mathrm{PET}}}}\;\times \;100$$

m_PET_ and m_PET-r_ represent the mass of initial PET and residual PET, respectively; M_DMT_ denotes the mass of DMT. M_PET_ and M_DMT_ correspond to the molecular weights of PET repeat units (192 g·mol⁻^1^) and DMT (194 g·mol⁻^1^), respectively.

### 2.4. Calculation of Environmental and Energy Impacts

Based on the literature, the environmental impact of PET depolymerization is evaluated by utilizing environmental factors and environmental energy influences. First, an energy economy coefficient (ε) is introduced to objectively compare the effects of parameters such as temperature, catalyst type, and feedstock ratio. Here, t denotes the reaction time (in minutes), T denotes the reaction temperature (in °C), and Y represents the yield of the monomer mass fraction defined in Equation (8). Sheldon et al. [35,36] refined the environmental factor (E-factor) by incorporating the influence of material inputs contributing to waste generation. The environmental energy impact (ξ) results from the combination of these two factors. Optimal processes typically exhibit a high ε coefficient alongside low E-factor and ξ values.

### 2.5. Characterization

Nuclear Magnetic Resonance (NMR) Spectroscopy: ^1^H NMR and ^13^C NMR spectra were recorded on a (Berlin, Germany) Bruker AVANCE III HD 400 MHz superconducting nuclear magnetic resonance spectrometer using a DMSO-*d*₆ as the solvent.

Fourier Transform Infrared Spectroscopy (FTIR): FTIR spectral analysis was conducted using a (Waltham, MA, America) Thermo Scientific Nicolet iS50 FTIR spectrometer in the wavenumber range of 4000 to 500 cm⁻^1^.

Differential Scanning Calorimetry (DSC): The melting point of DMT was determined using a (New Castle, DE, America) TA Instruments Q2000 differential scanning calorimeter. The measurement was conducted over a temperature range of 40 to 200 °C at a heating rate of 10 °C/min.

Transmission and Reflection Polarizing Light Microscope (PLM): The UPT103i transmission and reflection polarizing light microscopy system from (Chongqing, China) Youpu Optoelectronic Technology Co., Ltd. was used to observe the cross-sections of PET bottle chips before and after swelling.

Gas Chromatograph (GC): The analysis was conducted using a GC9790-II instrument manufactured by (Taizhou, China) Fuli Corporation, equipped with an RB-5 quartz capillary column (30 m in length, 0.32 mm inner diameter, 0.25 μm film thickness). High-purity nitrogen was employed as the carrier gas. For sample preparation, the analytes were dissolved in methanol, filtered through a 0.45 μm organic membrane, and then injected into the GC. The chromatographic conditions were set as follows: the injector temperature and detector temperature were both maintained at 300 °C. The column temperature was programmed with an initial hold at 80 °C for 2 min, followed by a ramp of 15 °C per minute to 250 °C and held for 10 min. Nitrogen served as the carrier gas, and a manual injection volume of 2.0 μL was used with a split ratio of 20:1.

## 3. Results and Discussions

### 3.1. Evaluation of Cosolvent Selection and Its Effectiveness

Table 1 presented the specific solubility parameter values of GVL, DMSO, THF, methanol, and PET [37]. The data show that GVL, DMSO, and THF exhibit more significant advantages in solubility parameter (δt) matching with PET compared to methanol. Specifically, methanol has a δt value of 27.1, differing by 5.2 from PET’s δt value (21.9), while GVL, DMSO, and THF have δt values of 19.5, 26.7, and 19.4, with differences from PET of only 2.4, 4.8, and 2.5, respectively. Among them, the solubility parameters of GVL and THF are closer to those of PET. However, due to its low boiling point (66 °C), THF tends to increase system pressure under high-temperature conditions, imposing higher requirements on reaction equipment. Therefore, GVL—a non-toxic biomass solvent with solubility parameters close to those of PET—was selected as the cosolvent. From a molecular interaction mechanism perspective, the five-membered lactone ring structure in GVL molecules can form strong intermolecular interactions with the aromatic regions in PET repeat units, bringing their solubility parameters closer. This effectively promotes the penetration of solvent molecules into PET polymer segments and significantly enhances the swelling and permeation capabilities of the solvent system toward PET. Furthermore, since PET’s solubility parameter lies between those of GVL and methanol, the solubility parameter of a mixed solvent composed of these two components can closely match that of PET.

Building upon this foundation, a GVL/methanol binary gradient mixed solvent system was further developed. Through precise regulation of the mixed solvent composition ratio, a synergistic depolymerization mechanism can be achieved. Among them, methanol, as a protic solvent, under the action of the catalyst, has its hydroxyl group (-OH) act as a nucleophilic reagent to directionally attack the carbonyl carbon of the ester bond (C=O) in the PET molecular chain under high-temperature conditions, thereby triggering the main chain breakage and generating DMT monomers. GVL, as an aprotic polar solvent, promotes the swelling of the PET chain segment through molecular permeation, effectively reducing the intermolecular forces within the macromolecular chains and facilitating the diffusion and permeation of the mixed solvent into the material. The synergy between these two solvents creates a gradient dissociation effect: the swelling effect of GVL significantly increases the accessible surface area of PET, providing more reaction sites for the catalyst and methanol with PET, thereby creating an optimal environment for the nucleophilic attack by methanol. Moreover, by finely tuning the ratio of GVL to methanol, it is possible to optimize reaction kinetics parameters, thus achieving a more pronounced enhancement in the depolymerization rate.

**Figure 1:**
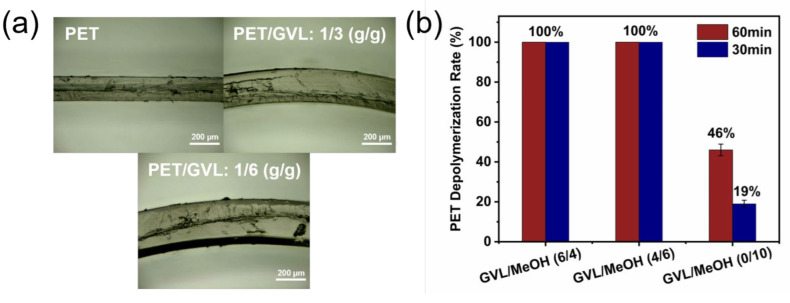
(**a**) Swelling effect of GVL on PET. (**b**) Effect of cosolvents on the depolymerization efficiency of PET in methanol.

To validate the above hypothesis, swelling tests were conducted with mass ratios of PET to GVL set at 1:3 and 1:6, respectively. Under a constant temperature condition of 150 °C, the swelling effect of GVL on PET bottle chips was observed via a polarizing microscope, as shown in Figure 1a. The test results indicate that with the increase in the proportion of GVL, the cross-sectional size of PET significantly increases. This phenomenon demonstrates that GVL can effectively induce swelling in PET, leading to the expansion of the material’s cross-sectional area, which verifies the swelling effect of GVL on PET. Based on the above findings, we employed a GVL/methanol mixed solvent in the conventional methanolysis process of PET to validate its efficient depolymerization capability. The experimental results are presented in Figure 1b. At a fixed temperature of 180 °C, when only methanol is used as the solvent, the PET depolymerization rate progresses slowly. After half an hour, the PET depolymerization rate was merely 19%; even after one hour of reaction, the dissociation rate only reached 46%. However, upon introducing γ-valerolactone (GVL) as a cosolvent into the system, the depolymerization efficiency of PET was significantly improved. Regardless of whether the mass ratio of GVL to methanol was 6:4 or 4:6, PET achieved complete depolymerization within 30 min. These preliminary experimental findings suggest that the GVL co-solvent exhibits a pronounced promoting effect on the methanolysis of PET.

### 3.2. Effect of Temperature on Depolymerization Effect

**Figure 2:**
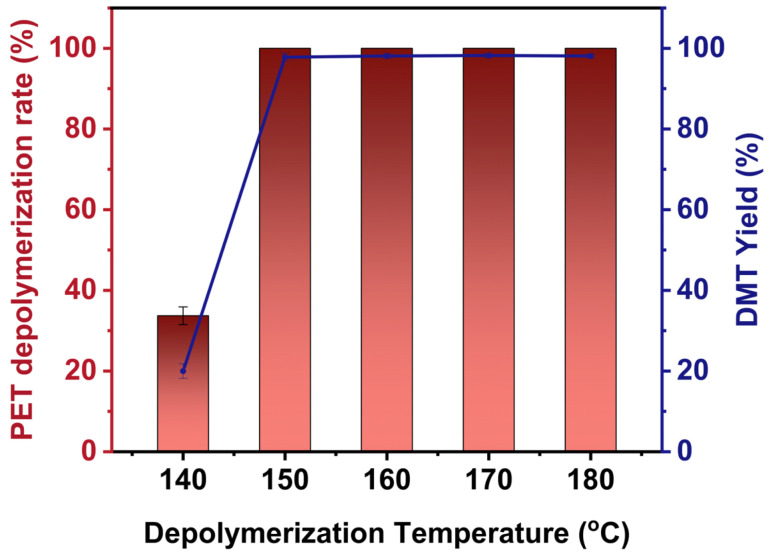
Effect of temperature variation on DMT yield and PET depolymerization efficiency.

To systematically investigate the regulatory mechanism of alcoholysis kinetics of PET in the GVL/methanol mixed solvent system, an in-depth analysis was performed to evaluate the effects of various reaction conditions on the depolymerization efficiency of PET and the yield of r-DMT. Firstly, the influence of temperature on the depolymerization process was systematically examined. As illustrated in Figure 2, the efficiency of PET depolymerization is strongly correlated with the reaction temperature. Specifically, when the reaction temperature is maintained at or above 150 °C, complete PET depolymerization (a depolymerization rate of 100%) can be achieved within 2 h, with the DMT yield remaining consistently over 98%. Notably, a significant drop in PET depolymerization efficiency is observed when the temperature decreases to 140 °C, resulting in a PET depolymerization rate of only 34% and a DMT yield of approximately 20% within the same time frame. The above results demonstrate that in the GVL/methanol mixed solvent system, the depolymerization temperature can be decreased to 150 °C. Moreover, the pressure of the reaction system at 150 °C is only 0.9 MPa, significantly lower than the 2–4 MPa of the conventional methanolysis process. Approximately 150 °C serves as a critical threshold for kinetic regulation, which not only overcomes the thermodynamic equilibrium limitations of the system but also ensures that the depolymerization reaction operates within its optimal kinetic range. The identification of this critical transition phenomenon offers a significant theoretical foundation for the optimization of the depolymerization process.

The regulation of temperature on depolymerization efficiency can be attributed to the following reasons: First, an increase in temperature enhances the mobility of polymer segments, leading to a free volume effect. This significantly improves the penetration and swelling of GVL molecules into the amorphous regions of PET, thereby facilitating the diffusion of methanol and catalysts to the active sites of ester bonds in the presence of GVL. Second, elevated temperatures enhance the nucleophilic attack activity of methanol. Above the critical temperature of 150 °C, the activation energy barrier for ester bond cleavage is overcome, markedly improving depolymerization efficiency. Notably, within the temperature range of 150–180 °C, the DMT yield does not fluctuate significantly with increasing temperature, suggesting that the rate-limiting step of the depolymerization reaction in this range may primarily involve the swelling and mass transfer process of PET.

It is worth noting that while increasing the temperature can significantly enhance the reaction rate, in the methanolysis of PET, a rise in temperature leads to a sharp increase in the vapor pressure of methanol within the closed reaction system. This not only imposes higher requirements on the pressure resistance of the reaction equipment but also substantially elevates the risks associated with experimental operations. Taking into account equipment safety, energy consumption costs, and operational feasibility, lowering the dissociation temperature holds positive significance. It can broaden the safety margin of the equipment, contribute to reducing energy consumption costs, and enhance operational feasibility.

### 3.3. Effect of Varying GVL/Methanol Ratios on the Efficiency of PET Depolymerization

**Figure 3:**
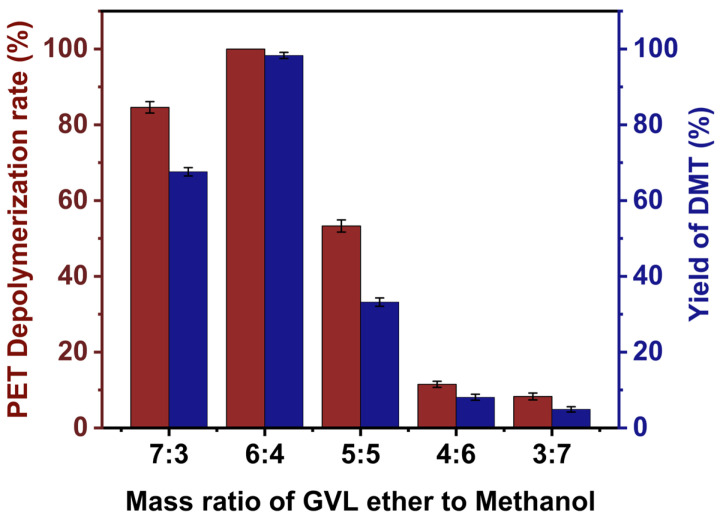
Effect of co-solvent to solvent ratio on DMT yield and PET depolymerization rate.

Furthermore, we systematically investigated the influence of the ratio of co-solvent (GVL) to depolymerizing agent (MeOH) on depolymerization efficiency within a mixed solvent system. As shown in Figure 3, under the conditions of 150 °C, 0.2 wt% Zn(OAc)_2_ content, and a material ratio of 1:10, the depolymerization rate of PET and the yield of DMT exhibit different trends at different solvent ratios. Figure 3 illustrates the trends in the PET depolymerization rate and DMT yield under varying solvent ratios at 150 °C. It is evident that as the GVL content in the mixed solvent system increases, the depolymerization efficiency of PET and the DMT yield initially increase before subsequently decreasing. At low GVL concentrations, the insufficient swelling effect on PET prevents the full stretching of PET molecular chains, making it challenging for the catalyst and depolymerizer to access sufficient ester bond sites, thereby limiting depolymerization efficiency. When the mass ratio of GVL to methanol reaches 6:4, the system achieves optimal PET depolymerization efficiency and DMT yield. At this point, the solubility parameters of the mixed solvent and the concentration of nucleophilic reagents reach an ideal balance, ensuring excellent compatibility with PET and adequate nucleophilic reagent availability. This promotes conformational extension of PET molecular chains via intermolecular forces, thereby fully exposing the previously encapsulated ester bond active sites for CH_3_OH nucleophilic attack. As the GVL content continues to rise, despite further enhancement of the swelling effect, the reduced concentration of nucleophiles leads to a decline in PET depolymerization efficiency. Nevertheless, the reaction system remains highly reactive compared to systems with lower GVL content.

### 3.4. Effect of Catalyst Concentration on the Efficiency of PET Depolymerization

**Figure 4:**
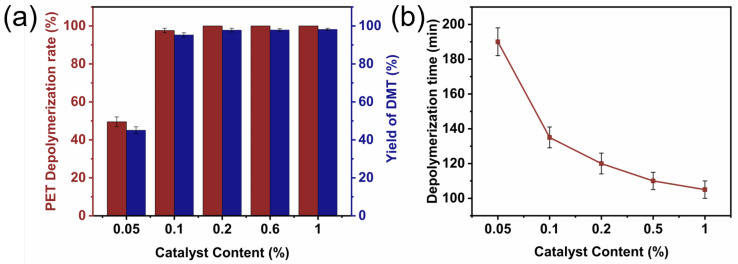
(**a**) Effect of catalyst content on PET depolymerization efficiency and DMT yield (**b**) Effect of catalyst content on reaction time under complete depolymerization of PET.

Further research was conducted on the effect of catalyst dosage on the depolymerization process under conditions of a reaction temperature at 150 °C and a GVL/CH_3_OH ratio of 6:4. As shown in Figure 4a, the concentration of Zn(OAc)_2_ has a significant impact on depolymerization efficiency. With increasing catalyst content, the depolymerization efficiency improves markedly. In the GVL/CH_3_OH system, when the catalyst dosage increased from 0.05% to 0.1%, the depolymerization rate of PET within 2 h rose significantly from 49.5% to 97.6%, with the DMT yield also increasing synchronously from 45.1% to 95.3%. This clearly demonstrates the activation effect of catalyst concentration on reaction activity. When the catalyst concentration reaches 0.2% or higher, PET can be completely depolymerized within 2 h, with the DMT yield maintaining above 97.8%. However, further increases in catalyst concentration may accelerate the depolymerization process but could result in higher costs and potentially adverse effects on DMT purity.

When the catalyst concentration reaches 0.2% or higher, as shown in Figure 4b, PET can be completely depolymerized within 2 h. Although further increasing the catalyst concentration may slightly accelerate the depolymerization process, this will undoubtedly increase costs. To clarify the specific impact of increased catalyst content on PET depolymerization efficiency, we conducted experiments under the condition of fixing the complete depolymerization of PET. The results show that when the catalyst content exceeds 0.2 wt%, the time required for complete depolymerization does not significantly shorten. This phenomenon is mainly caused by two factors: first, after the catalyst reaches a certain dosage, the active sites are fully occupied, and the additionally added catalyst cannot further exert its catalytic effect; second, the diffusion performance of reactants becomes a key factor restricting the improvement of depolymerization efficiency. In summary, when the catalyst content reaches 0.2 wt%, further increasing the dosage has a limited effect on improving the reaction efficiency; instead, it increases the experimental cost and the difficulty of post-processing impurity removal. Therefore, 0.2 wt% is selected as the optimal catalyst content.

### 3.5. Effect of Solid–Liquid Ratio on the Efficiency of PET Depolymerization

**Figure 5:**
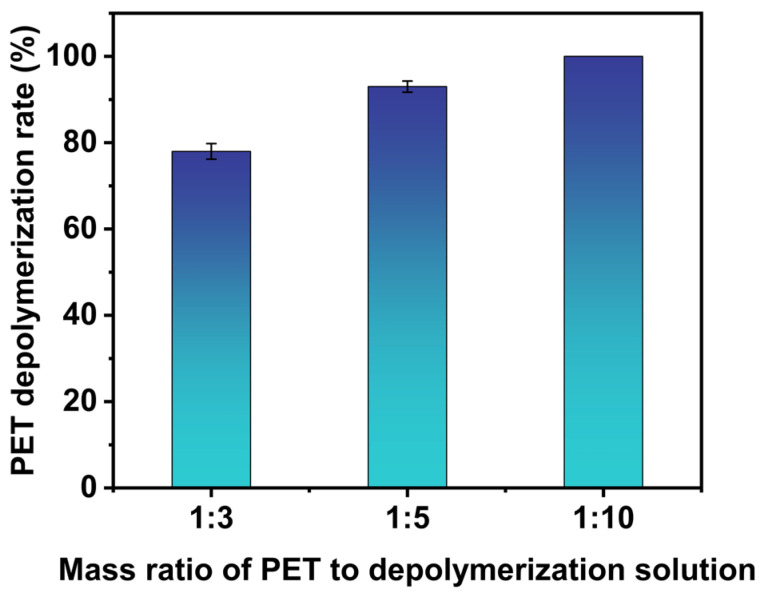
Effect of the mass ratio of PET to depolymerization medium on depolymerization rate.

In addition, the influence of the mass ratio of depolymerization solvent to PET on depolymerization efficiency was investigated. Under fixed conditions of reaction temperature at 150 °C, GVL/methanol mass ratio of 6:4, Zn(OAc)_2_ addition of 0.2 wt%, and reaction time of 2 h, experiments were conducted with varying PET/depolymerization solvent ratios. The results presented in Figure 5 demonstrate that a significant enhancement in PET depolymerization efficiency can be achieved by increasing the mass ratio of the depolymerization solvent to PET. Specifically, when the mass ratio increased from 1:3 to 1:10, the PET depolymerization efficiency rose from 78% to 100% within 2 h. This phenomenon is closely associated with the relative content of GVL and methanol to PET. A higher GVL content improves the swelling and permeation effects on PET, while methanol, acting as a nucleophilic reagent, facilitates the cleavage of ester bonds through nucleophilic substitution. Increasing the dosage of methanol enhances the probability of nucleophilic attack on ester bonds, thereby promoting the forward movement of ester exchange reactions. Conversely, insufficient methanol content limits nucleophilic attack, which becomes a critical factor hindering the improvement of depolymerization efficiency. However, excessively increasing the mass ratio of depolymerization solvent to PET may lead to increased energy consumption and reduced catalyst concentration in the system, potentially adversely affecting the PET depolymerization process.

### 3.6. Kinetics of Depolymerization Reactions

**Figure 6:**
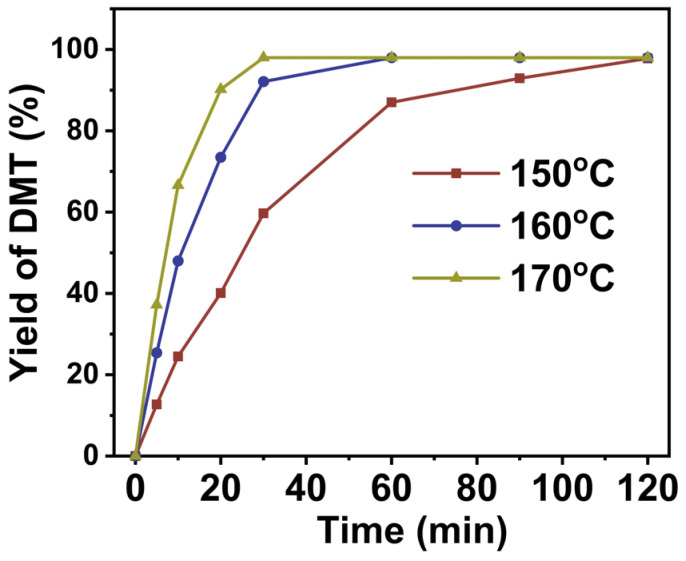
Effect of depolymerization time on DMT yield rate at different temperatures.

Based on the aforementioned studies regarding the influence of various reaction conditions on the depolymerization efficiency and DMT yield, the optimized process parameters for DMT yield were determined. The reaction kinetics of depolymerization in the GVL/methanol mixed solvent system under these optimized conditions were further investigated. As illustrated in Figure 6, the curves depicting the variation in DMT yield rate with reaction time at different temperatures are presented. It is evident that the depolymerization efficiency is highly sensitive to temperature. Specifically, at a reaction temperature of 150 °C, PET can be completely depolymerized within 120 min; when the temperature is elevated to 160 °C, the depolymerization time decreases to 60 min; further increasing the temperature to 170 °C allows the depolymerization process to be completed within merely 30 min.

**Figure 7:**
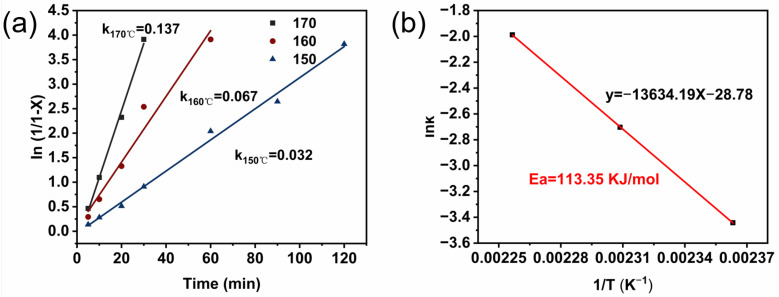
(**a**) Kinetics of PET depolymerization and (**b**) Arrhenius diagram of PET depolymerization activation energy.

Based on the data obtained at various temperatures and reaction times, linear fitting was employed to construct curves representing the relationship between DMT yield rate and reaction time at different temperatures [38]. The reaction rate constant (k) at each temperature was subsequently calculated. As illustrated in Figure 7, the reaction rate constant (k) exhibits a significant increase with rising temperature, with values increasing as follows: k_150 °C_ = 0.032, k_160 °C_ = 0.067, and k_170 °C_ = 0.137, indicating a temperature-dependent enhancement of reaction rate. Kinetic analysis reveals that the PET depolymerization process adheres to first-order reaction kinetics in the GVL/methanol system. Further fitting of the aforementioned data using the Arrhenius equation yielded an apparent activation energy (E_a_) of 113.35 kJ/mol for PET depolymerization within this system.

### 3.7. Analysis of Depolymerization Products

**Figure 8:**
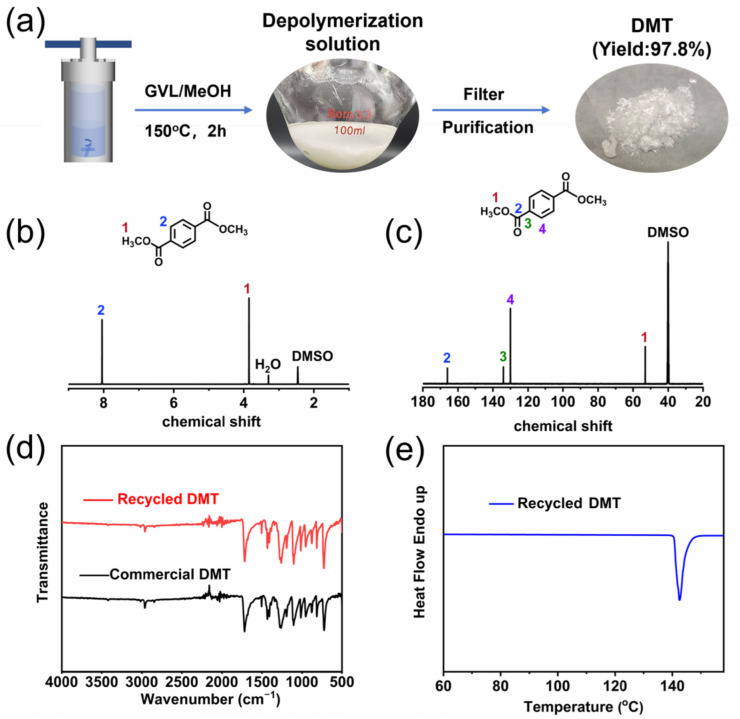
(**a**) GVL/Methanol depolymerization process (**b**) 1H NMR diagram of depolymerization products (**c**) 13C NMR diagram of depolymerization products (**d**) FTIR diagram of depolymerization products (**e**) DSC diagram of depolymerization products.

Figure 8a demonstrates the complete process flow of PET depolymerization in the GVL/methanol solvent system under the optimized conditions. The yield of DMT in this process is remarkably high, reaching 97.8%. Furthermore, the chemical structure and purity of the resulting DMT were comprehensively characterized using FTIR, NMR, and DSC. In the ^1^H NMR spectrum (Figure 8b), the single peak at 3.72 ppm is attributed to the proton resonance of the methyl group, while the sharp singlet at 8.13 ppm corresponds to the resonance signals of the four equivalent protons on the benzene ring. ^13^C NMR spectrum (Figure 8c) further validates the product structure at the atomic level: a signal at 53 ppm corresponds to the characteristic resonance of methyl carbon (-CH_3_), and a strong peak at 166 ppm falls within the typical chemical shift range for carbonyl carbon (C=O) in ester bonds. Resonance signals at 134 ppm and 130 ppm are attributed to the carbons directly bonded to the ester group and their vicinal carbons in the benzene ring, respectively. No additional impurity peaks were observed in the ^1^H NMR and ^13^C NMR spectrum, confirming the absence of unbroken oligomers or by-products in the depolymerization product. This result is consistent with DMT, as no abnormal peaks appear, fully demonstrating the purity and structural specificity of the product.

**Figure 9:**
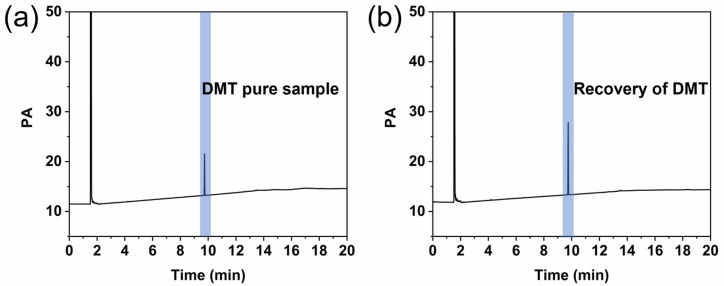
(**a**) Gas chromatogram of the commercial DMT sample. (**b**) Gas chromatogram of the recycled DMT.

The FTIR spectrum (Figure 8d) provides definitive evidence of functional groups. The strong absorption band at 1720 cm^−1^ arises from the symmetric stretching vibration of the C=O bond in the ester group, while the dual peaks at 1260 cm^−1^ and 1110 cm^−1^ correspond to the asymmetric and symmetric stretching vibrations of the C-O bonds in the ester group, respectively, with an intensity characteristic of typical ester compounds. The strong absorption band at 729 cm⁻^1^ originates from the out-of-plane bending vibration (C-H deformation vibration) of the benzene ring. These characteristics perfectly match the standard FTIR spectrum of commercial DMT. Additionally, the differential scanning calorimetry (DSC) curve reveals that the product displays a single, sharp melting peak at 142 °C, with a narrow melting range of only 1.5 °C. As shown in Figure 9, a comparison of the gas chromatograms of commercial pure DMT and recycled DMT reveals that the peak position of commercial DMT is 9.738, while that of recycled DMT is 9.748. The retention times of the two are nearly identical. Additionally, apart from the solvent peak, no other impurity peaks are observed in either chromatogram. This indicates that the product possesses a uniform composition and exhibits high purity.

### 3.8. Calculation of Environmental Energy

Drawing on the green chemistry assessment framework proposed by Sheldon et al. [34,35], a comprehensive quantitative evaluation of the PET depolymerization process was conducted using three key indicators: energy efficiency (ε), environmental impact (E), and energy-related environmental effects (ξ). As shown in Table 2, the GVL/methanol co-solvent system demonstrated superior sustainability characteristics. The energy economy index ε reached 5.4 × 10^−4^, attributable to the synergistic effects of GVL in enhancing methanolysis efficiency and achieving a high DMT yield of 98.7%. Environmentally, the system exhibited an exceptionally low E factor of 1.01, coupled with a significantly reduced ξ value of 1870. These results confirm the system’s advantages in energy-efficient utilization and environmental compatibility. Aligned with green chemistry principles requiring high ε values alongside minimized E and ξ parameters, this optimized process successfully balances high conversion efficiency with controlled energy consumption and waste generation, representing a progressive approach toward sustainable chemical engineering practices.

## 4. Conclusions

This work employed Hansen’s solubility parameter theory to identify γ-valerolactone (GVL), a bio-based green solvent, as a co-solvent for enhancing the methanolysis system of PET. Through systematic parameter optimization, the optimal reaction conditions were established as follows: GVL/methanol mass ratio of 6:4, reaction temperature of 150 °C, zinc acetate (Zn(OAc)_2_) catalyst loading of 0.2 wt%, and PET/mixed solvent mass ratio of 1:10. Under these conditions, PET can achieve 100% depolymerization efficiency within 2 h, with the reaction pressure decreased to 0.9 MPa and a DMT recovery rate of 97.8%. Consequently, the incorporation of GVL into the methanolysis system markedly enhances the compatibility with PET, enabling efficient depolymerization under relatively mild temperature conditions. Compared with traditional high-temperature methanol decomposition processes, this system demonstrates remarkable advantages in terms of depolymerization efficiency, moderate reaction conditions, environmental performance, and energy consumption, thereby showcasing broad application potential.

## Figures and Tables

**Table 1:** Solubility parameters of solvents.

	GVL	DMSO	THF	MeOH	PET
δ_d_	16.8	18.4	16.8	15.5	18.2
δ_p_	5.1	16.4	5.7	13.3	7.3
δ_h_	8.5	10.2	8.0	18.5	7.9
δ_t_	19.5	26.7	19.4	27.1	21.9

**Table 2:** Effects of alkaline hydrolysis of PET on environmental energy.

T (°C)	Reaction Time (min)	Catalyst	Solvent/PET(w/w)	Cat/PET (w/w)	Yield (%)	ε (°C^−1^ min^−1^)	E_factor_ (a.u)	ξ (°C min)	Ref.
200 °C	120	BLA	8.3:1	0.21	78	3.25 × 10^−5^	1.32	40,615	[25]
200 °C	30	MgO/NaY	6:1	0.04	91.0	1.5 × 10^−4^	0.70	4667	[23]
150 °C	120	Zn^2+^	10:1	0.002	97.8	5.4 × 10^−4^	1.01	1870	This work

## Data Availability

The original contributions presented in this study are included in the article. Further inquiries can be directed to the corresponding author.
